# Generation of Live Offspring from Vitrified Mouse Oocytes of C57BL/6J Strain

**DOI:** 10.1371/journal.pone.0058063

**Published:** 2013-03-13

**Authors:** Natsuki Kohaya, Katsuyoshi Fujiwara, Junya Ito, Naomi Kashiwazaki

**Affiliations:** 1 Laboratory of Animal Reproduction, School of Veterinary Medicine, Azabu University, Sagamihara, Japan; 2 Graduate School of Veterinary Sciences, Azabu University, Sagamihara, Japan; IGBMC/ICS, France

## Abstract

In mammals, unfertilized oocytes are one of the most available stages for cryopreservation because the cryopreserved oocytes can be used for assisted reproductive technologies, including *in vitro* fertilization (IVF) and intracytoplasmic sperm injection. However, it has generally been reported that the fertility and developmental ability of the oocytes are reduced by cryopreservation. C57BL/6J mice, an inbred strain, are used extensively for the production of transgenic and knockout mice. If the oocytes from C57BL/6J mice can be successfully cryopreserved, the cryopreservation protocol used will contribute to the high-speed production of not only gene-modified mice but also hybrid mice. Very recently, we succeeded in the vitrification of mouse oocytes derived from ICR (outbred) mice. However, our protocol can be applied to the vitrification of oocytes from an inbred strain. The aim of the present study was to establish the vitrification of oocytes from C57BL/6J mice. First, the effect of cumulus cells on the ability of C57BL/6J mouse oocytes to fertilize and develop *in vitro* was examined. The fertility and developmental ability of oocyte-removed cumulus cells (i.e., denuded oocytes, or DOs) after IVF were reduced compared to cumulus oocyte complexes (COCs) in both fresh and cryopreserved groups. Vitrified COCs showed significantly (*P*<0.05) higher fertility and ability to develop into the 2-cell and blastocyst stages compared to the vitrified DOs with cumulus cells and vitrified DOs alone. The vitrified COCs developed to term at a high success rate, equivalent to the rate obtained with IVF using fresh COCs. Taken together, our results demonstrate that we succeeded for the first time in the vitrification of mouse oocytes from C57BL/6J mice. Our findings will also contribute to the improvement of oocyte vitrification not only in animals but also in clinical applications for human infertility.

## Introduction

As one of the most informative experimental animals, mice have been used in a broad range of research fields. A large number of mutant or transgenic mouse lines have been produced by the microinjection of foreign DNA (see the review by Palmiter and Brinster [1]), intracytoplasmic injections of sperm-attached DNA [2], and infection with lentivirus [3] or *N*-ethyl-*N*-nitrosourea (ENU) mutagenesis [4]. In addition, 'gene-targeted' knockout mice have been generated via embryonic stem (ES) cells, spermatogonial stem cells [5], and DNA-nicking nucleases [6]. To manage this ever-increasing number of mouse lines, animal facilities will benefit greatly from cryopreservation of the germ cells.

Whittingham and his colleagues [7] first succeeded in the cryopreservation of mammalian embryos at −196°C by ice-seeding and a cryopreservation method that has been referred to as 'slow-freezing.' Rall and Fahy [8] also succeeded in the preservation of mammalian embryos at −196°C by an alternative method called the 'vitrification method.' In contrast to the slow-freezing method, the major advantage of the vitrification method is the elimination of the physiological damage caused by intracellular or extracellular ice crystal formation, and the reduction of chilling damage by shortening the exposure to suboptimal temperature [9]. The vitrification method is also simpler and quicker than the slow-freezing method because the embryos are out of the incubator for less than several minutes in the vitrification method, whereas with the slow-freezing method the equilibration alone takes more than 20 min [10]. Thus, vitrification can supplant slow-freezing as the optimal method for embryo cryopreservation in mammals.

Although the cryopreservation of embryos has improved with the development of robust vitrification protocols, the cryopreservation of oocytes is a developing technology. The first successful cryopreservation of mammalian oocytes by the slow-freezing method was reported in the 1970s [11], [12] and then reported in humans a decade later [13]. Much research has focused on the cryopreservation of mammalian oocytes by slow-freezing or subsequently with the vitrification method, resulting in live offspring in several mammalian species, including the rabbit [14], cattle [15], horse [16], and humans [17]. However, the overall success rate of oocyte cryopreservation is still lower than that of its unfrozen counterparts, even in mice.

Mouse oocytes were cryopreserved with an injection of trehalose at a high success rate [18], but this protocol requires a great deal of skill, making it difficult to use for the mass-cryopreservation of oocytes. Although it has been reported that treatment with trehalose also improves the vitrification of mouse oocytes, only 27.3% of vitrified oocytes were developed to term even under optimal conditions [19]. Thus, a more simple, efficient, and general-purpose protocol is in demand.

In the general procedure for oocyte cryopreservation in mammals, cumulus cells are removed from cumulus-oocytes complexes (COCs), and the denuded oocytes are then cryopreserved [18], [20]. Although it is acknowledged that cumulus cells have roles in oocyte maturation, some studies demonstrated that cumulus cells are also important for fertilization [21]–[24]. For example, disruption of pentraxin 3, which is a gene involving the conformation of COC, causes morphologically abnormal COC formation and reduces fertility *in vivo* [21], [22]. Moreover, some previous publications have shown that factors secreted from COCs *in vitro* are required to attract sperm to the COC, and for COC compaction by cumulus extracellular matrix assembly, sperm capacitation, and the enhancement of fertilization in mice [23], [24]. These results suggest the possibility that low fertility may be due to the removal of cumulus cells from the oocytes before cryopreservation. If so, the cryopreservation of oocytes with cumulus cells may lead to improved fertility without an additional method such as trehalose treatment.

We recently demonstrated the efficient vitrification of mouse oocytes that were surrounded by cumulus cells [25]. However, in that study, we tested outbred (ICR) mice but not inbred (C57BL/6) mice. In the present study we evaluated the *in vitro* effect of cumulus cells and cryoprotectants in vitrification medium on the cortical granule exocytosis, fertility and developmental ability of vitrified oocytes from C57BL/6J mice.

## Materials and Methods

All chemicals and reagents were purchased from the Sigma-Aldrich Corporation (St. Louis, MO, USA) unless otherwise stated. The study was approved by the ethical committee for vertebrate experiments at Azabu University (ID#197110325-1) [26].

### Animals

We used Crlj: C57BL/6J females (4–5 weeks old) for the collection of metaphase II (MII) oocytes and Crlj: C57BL/6J and Crlj: BDF1 males (12–24 weeks old) for sperm collection. The mice were purchased from Charles River Laboratories Japan (Yokohama, Japan). Mature female ICR mice (12–14 weeks old) were used as recipients for embryo transfer. Vasectomized male ICR mice (20–30 weeks old) were used to induce pseudopregnancies. The mice were housed in an environmentally controlled room with a 12-h dark/12-h light cycle at a temperature of 23±2°C and humidity of 55±5% with free access to a laboratory diet and filtered water.

### Oocyte collection

Cumulus oocyte complexes (COCs) at the metaphase-II stage were collected from the oviducts of C57BL/6J female mice (4–8 weeks) that were superovulated by an intraperitoneal injection of 5 IU equine chorionic gonadotropin (eCG; Nippon Zenyaku Kogyo, Tokyo) followed by 5 IU human chorionic gonadotropin (hCG; Asuka Pharmaceutical Co., Tokyo) 48 h later. Fourteen hours after the second injection, the females were sacrificed and their oviductal ampullae were removed. The oviductal ampullae were placed in oil, and COCs were collected from the oviductal ampullae with calcium- and magnesium-free modified PB1 (PB1(−)) [7] supplemented with 0.1% hyaluronidase. After the cumulus cells were removed, denuded oocytes (DOs) were washed 3 times with PB1(−) and used for further experiments. In some experiments, COCs were used for further experiments without the removal of cumulus cells.

### Vitrification of oocytes

In some experiments, COCs and DOs were used for vitrification. Vitrification was performed using a Cryotop device (Kitazato BioPharma Co., Shizuoka, Japan) as reported [27] with some modifications. In brief, COCs or DOs were placed in equilibrium solution [7.5% (v/v) ethylene glycol (EG), 7.5% (v/v) dimethylsulfoxide (DMSO), and 20% (v/v) fetal calf serum (FCS) in PB1(−)] for 3 min and then transferred into vitrification solution [15% (v/v) EG, 15% (v/v) DMSO, 20% (v/v) FCS, and 0.5 M sucrose in PB1(−)] for 1 min. Then 10–15 COCs or DOs were placed on a sheet of Cryotop in a small volume of the vitrification solution. The Cryotop was plunged into liquid nitrogen when the COCs or DOs were exposed to the vitrification solution for 1 min and then stored for at least 1 week. In the Cryotop method, vitrification solution is loaded with a narrow glass capillary onto the top of the film strip in a volume of less than 0.1 µl. After loading, almost all of the solution is removed to leave only a thin layer covering the oocytes [9].

The COCs or DOs were warmed by immersing the Cryotop in a warming solution composed of 0.5 M sucrose +20% FCS in PB1(−) at 37°C for 3 min, and then placed in 20% FCS PB1(−) at 37°C for 5 min. In some experiments, cumulus cells were also vitrified-warmed, as were COCs and DOs. The survival of the vitrified-warmed oocytes was morphologically evaluated. After being washed three times with TYH [28], COCs or DOs were transferred into a 100 µl drop of TYH and then used for IVF.

### *In vitro* fertilization

After dissections, the epididymides were removed and placed in a 35-mm sterile plastic dish containing 400 µl R18S3 medium [29]. The epididymal sperm were counted with a hematocytometer, and sperm motility and viability were evaluated as reported [30]. Namely, the sperm motility was assessed visually and determined by direct observation at 37°C under light microscopy at 100x. For the cryopreservation, spermatozoa were loaded into 0.25-ml plastic straws (Fujihira Industry, Tokyo). The straws were exposed to liquid nitrogen (LN) vapor (about −150°C) for 10 min and then plunged into LN and stored for at least 1 week. For thawing, the straws were kept in a 37°C water-bath for 10 sec and the contents were then expelled into a 35-mm sterile plastic dish.

Post-thaw sperm viability and motility were evaluated as described above. The frozen-thawed spermatozoa were resuspended in TYH medium, and the number and motility of the sperm were assessed as described above. Fresh and frozen-thawed spermatozoa were incubated for the induction of sperm capacitation in TYH for 2 h or 1 h, respectively. The sperm were then added into the TYH drops containing COCs, DOs or DOs with cumulus cells (the final sperm concentration was 2×10^6^ sperm/ml) and co-cultured for 6 h. After the culture, the DOs or COCs were transferred into a 100-ul drop of potassium simplex optimized medium (KSOM-AA) [31] supplemented with or without 0.1% hyaluronidase, respectively. The cumulus cells of COCs were then removed by gentle pipetting. The oocytes were washed three times in KSOM-AA and then evaluated for fertility using an inverted phase-contrast microscope (Olympus, Yokohama, Japan). Oocytes having two pronuclei were determined to be fertilized. Only fertilized oocytes were transferred into 100 µl of KSOM-AA and were cultured up to 120 h at 37.5°C under 5% CO_2_ in air. Cleavage and blastocyst formation of the oocytes were evaluated at 18 h and 114 h postfertilization, respectively.

### Embryo transfer

To evaluate the *in vivo* development of the IVF oocytes, we transferred putative embryos into the oviducts of recipients after the induction of pseudopregnancy as described [25]. Female mice used as recipients for embryo transfer were mated with vasectomized males on day 0 between 16:00 and 22:00 to induce pseudopregnancies. On day 1 between 21:00 and 22:00, nine to ten 2PN oocytes were transferred into each oviduct of the recipients. On the morning of day 20, the embryo recipient females underwent caesarean section to confirm the pregnancy and the normality of the offspring.

### Statistical Analyses

Each experiment had at least three replicates. More than 100 oocytes were used for each treatment group in this study, except for the embryo transfer. All percentage data were subjected to arcsine transformation before statistical analysis. Data were analyzed by one-way analysis of variance (ANOVA) and Tukey's test. *P*<0.05 was considered significant. Data are shown as means±standard error of means (S.E.M.).

## Results

**Figure 1:**
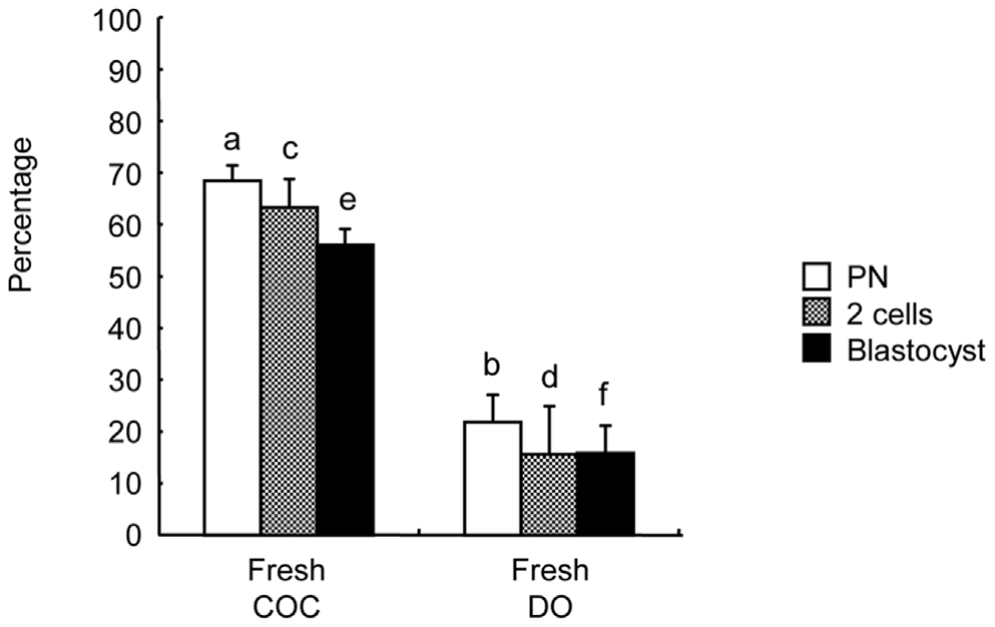
The effects of cumulus cells on the fertility and developmental ability of C57BL/6J mouse oocytes after IVF. Data are means±S.E.M. ^a-b,c-d,e-f^ Different superscripts denote a significant difference (*P*<0.05). In each treatment group, more than 100 oocytes were examined. PN, pronuclear stage; COC, cumulus oocyte complexes; DO, denuded oocytes.

**Figure 2:**
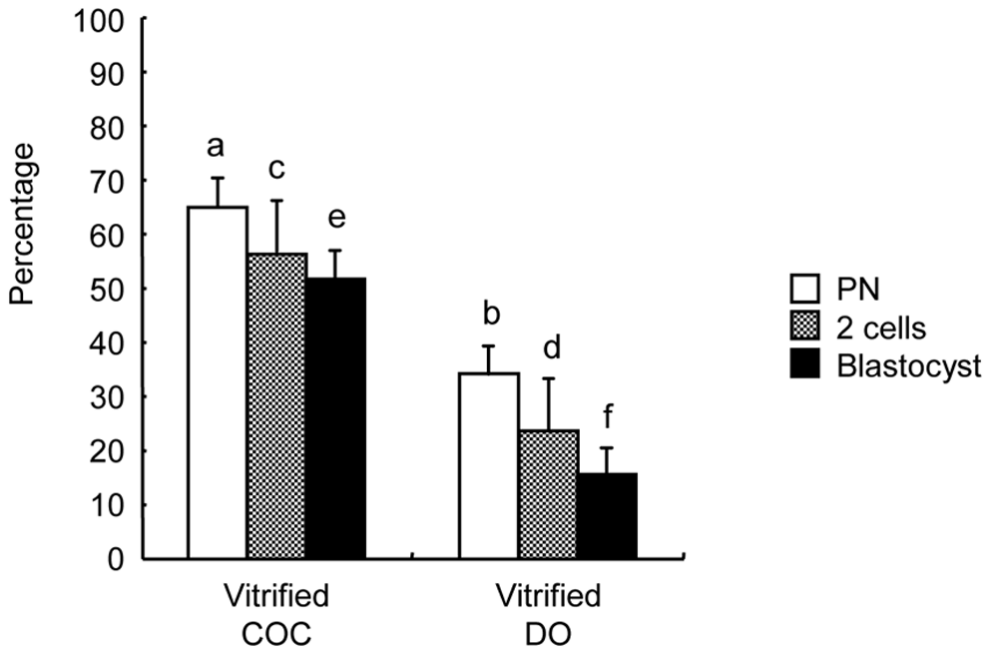
The effects of cumulus cells on the fertility and developmental ability of vitrified C57BL/6J mouse oocytes after IVF. IVF was carried out using frozen-thawed BDF1 mouse sperm. Data are means±S.E.M. ^a-b,c-d,e-f^ Different superscripts denote significant difference (*P*<0.05). In each treatment group, more than 100 oocytes were examined.

The IVF results of fresh COCs and DOs are shown in Figure 1. First, we used frozen-thawed sperm from BDF1 mice, which worked well for *in vitro* fertilization as reported [30]. Fresh COCs and DOs were cocultured with the frozen-thawed spermatozoa. The rates of fertilization, cleavage, and blastocyst formation in the fresh COC group were 67.5±1.4%, 63.6±1.8%, and 53.7±1.9%, respectively. In contrast, those in the fresh DO group were 23.6±2.6%, 19.6±5.7%, and 19.6±3.6%. These rates were significantly lower than those in the fresh COC group. Conversely, in the vitrified COC group, the rate of fertilization was high (62.1±3.2%) (Fig. 2). Most of the oocytes developed to the 2-cell stage (57.0±3.6%) and blastocysts (45.9±4.5%). In the vitrified DO group, these rates were low (33.3±6.1%, 24.8±4.9%, and 18.4±3.1%, respectively), and thus the rates in the vitrified COC group were significantly higher than those in the vitrified DO group (*P*<0.05).

**Figure 3:**
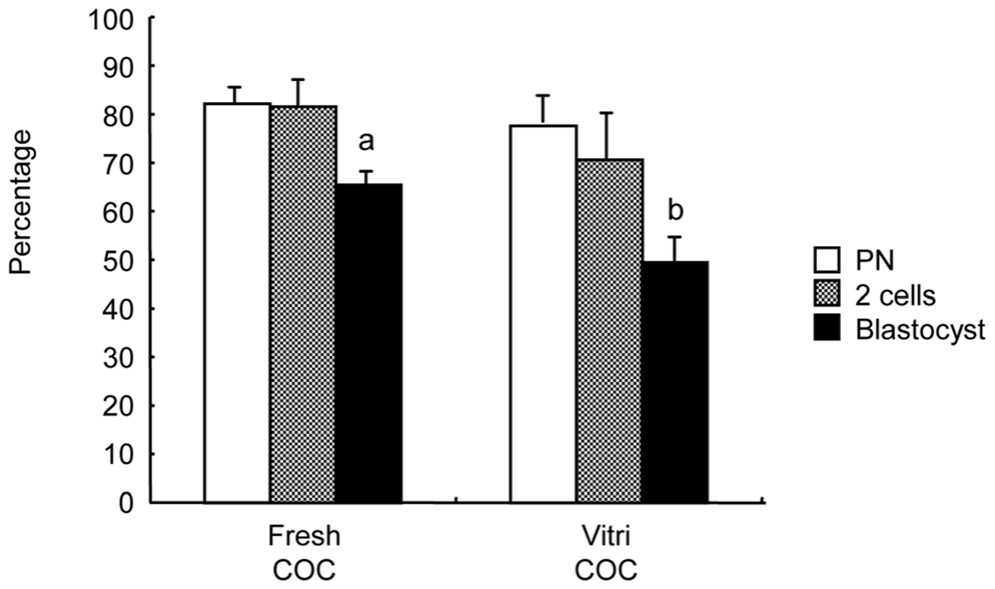
Fertility and developmental ability of vitrified-warmed cumulus oocyte complexes (COCs) after IVF in C57BL/6J mice. IVF was carried out using fresh C57BL/6J mouse sperm. Data are means±S.E.M. Different superscripts denote significant difference (*P*<0.05). In each treatment group, more than 100 oocytes were examined.

As the second experiment, we used fresh spermatozoa derived from C57BL/6J mice to produce inbred zygotes. The IVF results of the COCs are shown in Figure 3. In the fresh COC group, as expected, the rate of fertilization was high (81.7±1.4%). Most of the oocytes developed into 2-cell embryos (80.3±1.8%) and blastocysts (66.1±1.9%). In the vitrified COC group too, the fertilization rate was high (73.3±2.6%), equivalent to that in fresh COC group. Most of the oocytes developed to 2-cell embryos (66.7±5.7%) and blastocysts (43.0±3.6%). To confirm the *in vivo* development of the 2-cell embryos derived from vitrified COCs, we transferred the 2-cell embryos to pseudopregnant females. All transferred females that received embryos became pregnant, and 51 pups were obtained from vitrified COCs (Table 1). The offspring were visually normal. The rate of offspring obtained using vitrified COCs (56.7±3.6%) was similar to that using fresh COCs (57.8±2.3%), indicating that the successful vitrification of mouse oocytes has achieved.

**Table 1:** In vivo development of vitrified COCs from C57BL/6J mouse after in vitro fertilization.

Oocytes	Transferred embryos	Pregnant/recipients (%)	Litters/pregnancies (%)	Offspring (%)
Fresh	90	5/5 (100)	5/5 (100)	52 (57.8 ±5.6)
Vitrified	90	5/5 (100)	5/5 (100)	51 (56.7 ±2.3)

## Discussion

The successful cryopreservation of oocytes is highly desirable because it leads to a high efficiency of oocyte cryopreservation, making large numbers of viable oocytes available for the generation of offspring via IVF and intracytoplasmic sperm injection (ICSI). If the oocytes from C57BL/6J mice can be cryopreserved, the cryopreservation protocol will be useful for the high-speed production of not only gene-modified mice but also hybrid mice from different gene-modified mice. However, it is also well known that cryopreserved oocytes generally show low developmental ability after IVF, and thus technology such as ICSI is required for obtaining offspring in mice [20]. Alternative methods such as the injection of trehalose into oocytes [18] or treatment with tehalose [19] were reported to improve the low tolerance of mouse oocytes to cryopreservation. Since these alternative methods require a great deal of skill and cannot be used for the cryopreservation of many oocytes at the same time, a more simple, efficient, and general-purpose protocol is in demand. The object of the present study was to establish the successful cryopreservation of mouse oocytes derived from the C57BL/6J strain using vitrification, which has been known as a simpler and quicker protocol [32].

Chemokines secreted from COCs induce sperm capacitation and enhance fertilization, providing evidence of a regulatory loop between sperm and COCs during fertilization [23]. It was also demonstrated that chemokine signaling facilitates both sperm attraction to COC and COC compaction by the cumulus extracellular matrix assembly [24]. Our present results show that DOs have low fertility after IVF, even fresh DOs (Fig. 1). When ICSI was applied to vitrified DOs, most of the DOs survived injection (>75%) and developed to the 2-cell stage (>85%), even in several inbred strains [20]. Park *et al*. [25] also demonstrated that more COCs are fertilized and develop to the 8-cell stage after vitrification and warming compared to DOs in mice, although the rate is still quite low. These results strongly suggest that cumulus cells are indispensable for the successful vitrification of mouse oocytes if these vitrified oocytes are used for IVF. The details underlying the role of cumulus cells in vitrified oocytes during IVF remain to be clarified in further studies.

The results from our present study are much superior to those obtained in outbred mice [18], [19], [33]. Sanchez-Partida *et al.* [19] reported that 39% of vitrified oocytes developed into blastocysts and only a few oocytes developed to term (13%) [18]. Eroglu *et al.* [18] also showed that the offspring rate derived from vitrified oocytes was 19% (4 pups of 21 embryos). In our previous study, most of the vitrified oocytes (75%) developed to the blastocyst stage and 92 pups were obtained from 134 transferred embryos (69%) [25].

Even in the C57BL/6 strain, our data showed a significant improvement of fertility of vitrified oocytes compared to study of Endoh et al [20]. The advantages of the present study are as follows. First is the faster cooling and warming rates, which are indispensable to the higher cryopreservation success rate. The increases in the cooling and warming rates is achieved by minimizing the volume of the vitrification solution [34]. Indeed, numerous devices or methods have been developed to achieve a small volume of vitrification solution. An electron microscope grid [35], a gel-loading tip [36], open pulled straws (OPS) [37], the CryoLoop [38], solid surface vitrification [39], microdrops [40], and nylon mesh [41] were developed to minimize the volume of the vitrification solution.

In the present study we used the Cryotop device, which has a thin strip of plastic film. Kuwayama *et al*. [42] described the Cryotop as the latest minimum volume vitrification approach (less than 0.1 µl), and they estimated the cooling-warming rate to be up to 40,000°C/min with the Cryotop because the oocytes or embryos are covered with only a very thin solution layer that is achieved by removing almost all medium before cooling. Although fertility and developmental abilities were not compared using different devices, it was shown that vitrification with the Cryotop yields higher post-warming survival than either a gel-loading tip or the CryoLoop in rabbit embryos [36].

A study using pigs also found that the Cryotop method is superior to the Open Pulled Straw technique for the vitrification of matured oocytes [43]. Moreover, we succeeded in carrying out the vitrification of pronuclear-stage embryos in rats using the Cryotop method, at a high success rate [27]. These results suggest that a small volume of vitrification medium contributed to the higher fertility and developmental ability of vitrified-warmed COCs in the present study, in contrast to the results of earlier studies [18], [19], [33]. Cumulus cells may also have a role in the protection of oocytes from the damage by cooling and warming, although this matter remains to be clarified.

Taken together, our present results demonstrate for the first time the successful vitrification of mouse oocytes from the C57BL/6J strain. This method does not require additional treatment, e.g., trehalose injection or treatment. Our vitrification protocol will be useful for the production of not only gene-modified mice but also hybrid mice from different gene-modified mice, at high speed.
